# Hypoxia‐induced polypoid giant cancer cells in glioma promote the transformation of tumor‐associated macrophages to a tumor‐supportive phenotype

**DOI:** 10.1111/cns.13892

**Published:** 2022-06-28

**Authors:** Yuyang Liu, Ying Shi, Mengwan Wu, Jialin Liu, Hong Wu, Chuan Xu, Ling Chen

**Affiliations:** ^1^ Medical School of Chinese PLA Beijing China; ^2^ Department of Neurosurgery Chinese PLA General Hospital Beijing China; ^3^ School of Medicine University of Electronic science and Technology of China Chengdu China; ^4^ Integrative Cancer Center& Cancer Clinical Research Center Sichuan Cancer Hospital Chengdu China

**Keywords:** glioma, hypoxia, polypoid giant cancer cell, tumor‐associated macrophages

## Abstract

**Aims:**

Polypoid giant cancer cells (PGCCs) represent a unique subgroup of stem‐like cells, acting as a critical factor in promoting the recurrence of various solid tumors. The effect of PGCCs on the tumor malignancy of glioma and its immune microenvironment remains unclear.

**Methods:**

Bioinformatic analysis was performed to investigate the relationship between M2 tumor‐associated macrophages (TAMs) infiltration and survival of glioblastoma (GBM) patients. The spatial location of M2 TAMs in GBM was also investigated using the Ivy Glioblastoma Atlas Project (Ivy GAP) database. PGCCs were quantified in glioma of different grades. CoCl_2_ was used to induce PGCCs in cultures of A172 cells. PGCCs, and their progeny cells in cultures were further evaluated for morphological features, tumorsphere formation, and TAMs activation.

**Results:**

The magnitude of M2 TAMs infiltration is significantly correlated with poor survival in GBM patients. M2 TAMs were enriched in the perinecrotic zone (PNZ) of GBM and positively correlated with hypoxic levels. Increased PGCCs were detected in glioma specimens of higher grades. CoCl_2_ induced hypoxia and the transformation of A172 cultures into PGCCs, producing the progeny cells, PGCCs‐Dau, through asymmetric division. PGCCs and PGCCs‐Dau possessed tumor stem cell‐like features, while PGCCs‐Dau enhanced the polarization of TAMs into an M2 phenotype with relevance to immunosuppression and malignancy in GBM.

**Conclusions:**

PGCCs promote malignancy and immune‐suppressive microenvironment in GBM. PGCCs or their progeny cells may be a potential therapeutic target for GBM.

## Graphical abstract

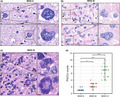

## 1 INTRODUCTION

Glioblastoma (GBM), the WHO grade IV glioma, is the most common and fatal type of primary brain cancer.^1^, ^2^, ^3^ Although multimodal treatments are available, including surgical resection, chemotherapy, and radiotherapy, the median survival of patients remains less than 16 months.^4^, ^5^ The tumor microenvironment plays a pivotal role in supporting malignant growth and progression. The tumor microenvironment within GBM consists of multiple components, including parenchymal cells, blood vessels, soluble factors, extracellular matrix, and infiltrating immune cells.^6^, ^7^, ^8^ Among them, tumor‐associated macrophages (TAMs) are enriched in GBMs and responsible for tumor invasion, cancer immunosuppression, and even therapy resistance.^9^

Recent studies suggested that TAMs could be polarized into two major phenotypes: M1 macrophages (tumor‐suppressive type) and M2 macrophages (tumor‐supportive type). While M1 TAMs promote phagocytosis, inflammation, and host immunity, M2 TAMs are immune‐suppressive and fuel cancer progression.^10^ Unfortunately, most TAMs display M2‐type properties in the GBM microenvironment.^4^ Although M2 TAMs are a proven driving force that promotes GBM malignancy, the mechanism underlying macrophage polarization into an M2 phenotype in GBM remains elusive.

In addition to cellular and bioactive components, hypoxia is also one of the most critical features of the glioblastoma microenvironment.^11^ Related studies showed that the hypoxic microenvironment modulates tumor immunogenicity, plasticity, and therapeutic resistance and promotes the invasion of tumor cells into the normal tissue, which forms a significant impediment to surgical resection, radiotherapy, and chemotherapy.^12^, ^13^, ^14^ The unique pathological features of GBM include necrotic foci with surrounding microvascular hyperplasia and cellular pseudopalisades. Investigations into the origin of pseudopalisades suggest that this distinctive structure is created by tumor cells that migrate away from the central hypoxic region and form an invading front.^12^ Glioma stem cells (GSCs) are a unique subset of tumor cells enriched in hypoxic niches and involved in tumor recurrence and invasion. Additionally, hypoxia plays a crucial role in stemness maintenance.^15^, ^16^

Polypoid giant cancer cells (PGCCs), defined as cancer cells whose nucleus is at least three times larger than the parental generation, compose a unique subtype of tumor cells.^17^ PGCCs commonly occur in advanced‐stage tumors and are associated with recurrence and poor prognosis.^18^ According to related studies, PGCCs have unique morphological characteristics and acquire hazardous properties of genomic instability, chemoresistance, radioresistance, and stemness.^19^ Moreover, these cells can produce progeny through a particular pattern called neosis.^20^ Mononuclear or multinuclear giant cells are one of the most prominent histopathological features of glioblastoma.^21^, ^22^ Due to the lack of giant cell samples, there were few studies on the phenotype and function of these particular cell subgroups in glioblastoma.

Previous studies have demonstrated that TAMs and PGCCs are enriched in the hypoxic regions.^4^, ^10^, ^18^ Interestingly, both PGCCs and TAMs populations are increased after treatment.^23^, ^24^ As significant prognostic indicators, the potential interaction between them needs to be further investigated in glioma.

## 2 MATERIALS AND METHODS

### 2.1 Bioinformatics analysis based on public databases

Clinical information and corresponding RNA‐seq data of 698 glioma samples were obtained from the TCGA database (https://portal.gdc.cancer.gov/). The RNA‐seq data of different anatomic regions of GBM was downloaded from the Ivy GAP database^25^ (https://portal.brain‐map.org/). The raw data were normalized using the fragment per kilobase of exon per million fragments mapped (FPKM) method, and log2 (FPKM+1) transformation was applied for the subsequent analyses. The infiltration levels of TAMs (M1 and M2) in TCGA glioma patients were evaluated by CIBERSORT, CIBERSORT‐ABS and QUANTISEQ Score directly downloaded from TIMER 2.0 database^26^, ^27^, ^28^ (http://timer.cistrome.org/). The hypoxic level in GBM was evaluated using the hypoxia metagene algorithm based on transcriptome data.^29^

### 2.2 Glioma sample collection

This research was approved by the Institutional Research Ethics Committee of the PLA General Hospital (batch number: S2018‐089‐01). Signed informed consents were collected from all participating patients. In this study, ten paraffin‐embedded glioma tissues, including 3 cases of WHO II, 3 cases of WHO III, and 4 cases of WHO IV, were processed for hematoxylin and eosin (HE) staining. At least two independent pathologists graded these tumors.

### 2.3 PGCCs quantitative analysis in glioma samples

Pathologically, PGCCs are defined as tumor cells with a nucleus at least three times larger than diploid cells (Zhang et al.).^18^ The maximal diameter of cell nuclei was measured using the CaseViewer software (2.4 version) for this study. For each case, five microscopic fields were assessed at × 200 magnification, and the number of PGCCs was recorded for further analysis.

### 2.4 Cell lines and cell culture

A172, the human glioma cell line (American Type Culture Collection), was cultured in DMEM supplemented with 10% fetal bovine serum, penicillin (100 units/ml), and streptomycin (100 μg/ml). THP1 cells (American Type Culture Collection) were cultured in RPMI 1640 supplemented with 10% fetal bovine serum, penicillin (100 units/ml), and streptomycin (100 μg/ml).

### 2.5 Induction and maintenance of A172‐PGCCs

The A172 cell lines were cultured in complete DMEM medium until the cells reached 90% confluence. At first, we treated the cells with 100 μm CoCl_2_ for 1d. After being rinsed with PBS, the cells were cultured in medium with FBS and antibiotics for recovering from CoCl_2_ treatment for 7d. With the increased CoCl_2_ concentration, more PGCCs survived, and almost all regular‐sized cells died (Figure 3).

### 2.6 Single‐cell cloning

Single‐cell cloning was performed to obtain the daughter cells of PGCCs. Briefly, A172‐PGCCs were washed twice with PBS and dissociated with 0.05% trypsin. Cell numbers were counted with trypan blue staining. Single‐cell cloning was carried out by limiting dilution, and the cells were seeded at one cell/well into a 96‐well plates. When each isolated clone was grown to 80% confluence, the cells were sequentially transferred into 24‐well plates, 6‐well plates, and 6 cm dishes. Finally, we obtained the daughter cells of PGCCs named A172‐PGCCs‐Dau (Figure 4).

### 2.7 Tumorsphere formation assays

Parental cells and progeny cells of each group were cultured with serum‐free stem cell medium (Dulbecco's Modified Eagle's Medium [DMEM]/F12 medium with 20‐ng/ml EGF, 20‐ng/ml basic fibroblast growth factor (bFGF), and 2% B27) in low‐attachment plates (Corning REF 3471). One thousand test cells in 3 ml of stem cell medium were cultured in low‐attachment dishes. Spheroid formation was then observed for an additional 7 days. Spheroids greater than 100 μm in diameter were counted.^30^

### 2.8 THP1‐derived M0 macrophage‐like cells and PGCCs‐educated macrophage

THP1 cells were primed with PMA (Peprotech, 100 ng/ml) to become unpolarized M0 macrophages‐like cells. After 48 h of stimulation, the medium was replaced by tumor conditioned medium, and cells were incubated for another 72 h.

### 2.9 Western blot

An equal amount of proteins (30 μg) were separated on 10% SDS‐PAGE gels and transferred onto PVDF membranes (0.45 μm; Amersham Bioscience). The membranes were incubated with primary antibodies overnight at 4°C and then with secondary antibodies for 2 h at room temperature. Immunoreactivity signals were amplified using the ECL Plus Western blotting detection system. The list of antibodies used in this study is presented in Table S1.

### 2.10 Quantitative real‐time PCR

Quantitative real‐time PCR (qRT‐PCR) was performed with a CFX96 Real‐Time PCR Detection System (Bio‐Rad). The expression of GAPDH was used for the normalization. The primers used in this study are presented in Table S2.

### 2.11 Conditioned medium preparation

Parental cells and progeny cells were cultured in DMEM supplemented with 10% fetal bovine serum for 3 h. Cells were washed three times with PBS and resupplied with serum‐free RPMI 1640 medium to prepare a serum‐free conditioned medium. Conditioned medium was collected from cultures at 4 × 10^5^ cells/ml density. The cells were removed by centrifugation (3500 rpm, 30 min), and the conditioned medium was sterile filtered through a 0.2 μm filter.

### 2.12 H&E staining

A172 and A172‐PGCCs were seeded in 24‐well culture plates plated with cell‐climbing slices. The cell‐climbing slices were then counterstained with hematoxylin for 1 min and eosin for 2 min. Finally, the cell‐climbing slices were placed on the glass slide and subjected to microscopic examinations.

### 2.13 Immunofluorescent staining

Slides with formalin‐fixed cultured cells or spheroids were incubated with 10% of goat blocking serum (AR0009, Boster) for 1 h, then diluted primary antibodies overnight at 4°C. Slides were further incubated with a fluorescence‐conjugated second antibody at room temperature for 2 h. Nuclei were counterstained with DAPI for 5 min. Cell morphology was analyzed with the PKH26 (MINI26‐1KT; Sigma‐Aldrich) and Phalloidin (CA1610; Solarbio), following the manufacturer's instructions. Images were obtained using a Nikon A1 laser confocal microscope.

### 2.14 Statistical analyses

Normality was assessed with Shapiro–Wilks test. Statistical differences were determined using the Mann–Whitney U rank sum test for two groups. Ordinary one‐way ANOVA and Kruskal–Wallis test were used for multiple comparisons. Tukey's multiple comparisons test (for ordinary one‐way ANOVA) and Dunn's multiple comparisons test (for Kruskal–Wallis test) were used to correct for multiple comparisons. Survival analysis of GBM patients was conducted for progression‐free interval (PFI), and the optimal cut‐off points with minimum *p*‐value was generated by the R package survminer (Version 0.4.9) automatically. The correlation between levels of TAM signature and hypoxic level in different anatomic regions of GBM was estimated using the spearman r test. Summary of correlation analyses are illustrated in the heatmap. All statistical analyses were performed by R software (Version 3.6.3) with statistical significance at *p < 0.05, **p < 0.01 and ***p < 0.001.

## 3 RESULTS

### 3.1 M2 TAMs predicted poor prognosis for GBM patients and enriched in the perinecrotic zone

**FIGURE 1:**
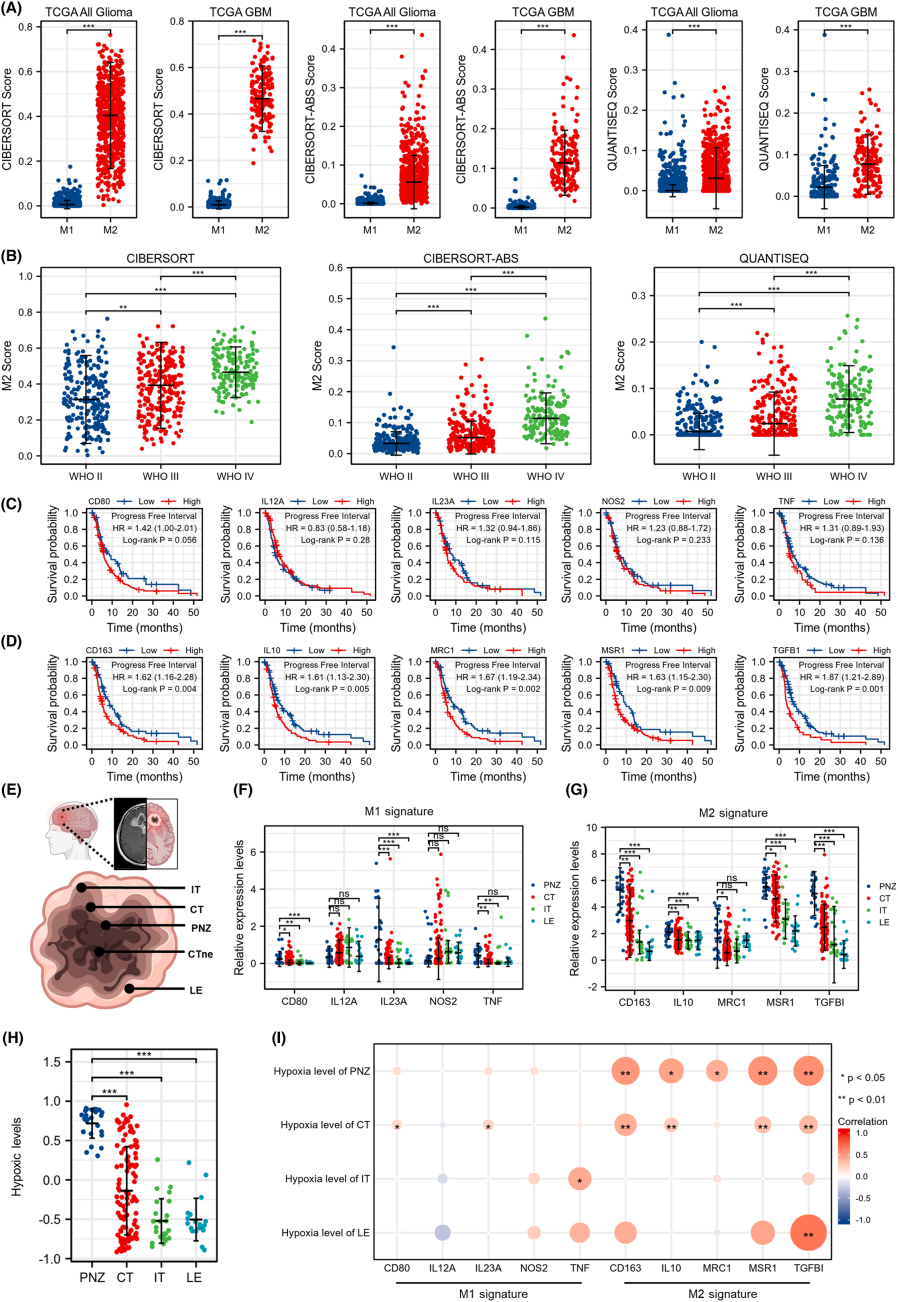
M2 TAMs is a prognostic biomarker for glioma and locates in PNZ. (A) CIBERSORT Score, CIBERSORT‐ABS Score and QUANTISEQ Score of TAMs (M1 and M2) in all glioma and GBM. Data are shown as median ± interquartile range, ***p < 0.001, Mann–Whitney U rank sum test. (B) CIBERSORT Score, CIBERSORT‐ABS Score and QUANTISEQ Score of M2 TAMs in glioma with different WHO grade. Data are shown as median ± interquartile range, **p < 0.01, *** *p* < 0.001, Kruskal–wallis test and Dunn's multiple comparisons test. (C, D) Kaplan–Meier survival analysis of TAMs (M1 and M2) signature expression and the progression‐free interval of GBM patients from the TCGA database, log‐rank test. (E) The anatomic regions of GBM according to the Ivy GAP database. (F, G) The relative expression levels of TAMs (M1 and M2) signature in different anatomic regions of GBM. Data are shown as median ± interquartile range, ns, *p* ≥ 0.05, *p < 0.05, **p < 0.01, *** *p* < 0.001, Kruskal–wallis test and Dunn's multiple comparisons test. (H) The hypoxic levels in different anatomic regions of GBM. Data are shown as median ± interquartile range, *** *p* < 0.001, Kruskal–wallis test and Dunn's multiple comparisons test. (I) Bivariate correlation analyses between the levels of TAMs (M1 and M2) signature and hypoxic levels in different anatomic regions of GBM. *p < 0.05, **p < 0.01, Spearman r test

According to CIBERSORT Score, CIBERSORT‐ABS Score, and QUANTISEQ Score, the infiltration level of M2 TAMs was significantly higher than M1 TAMs in all glioma and GBM (Figure 1A). In addition, M2 TAMs score was steadily increased with pathologic grade of glioma (Figure 1B). Survival analysis based on TAMs (M1 and M2) signature revealed that all the M2 signature but not the M1 signature were negatively correlated with the prognosis of glioma patients (Figure 1C and D). Based on the Ivy GAP database, the GBM tissue was divided into 5 parts (Figure 1E). Relative expression levels of TAMs signature demonstrated that some M1 signature (CD80 and IL23A) and M2 signature (CD163, IL10, MSR1, and TGFB1) were highest in the perinecrotic zone (PNZ) (Figure 1F and G). Moreover, hypoxic levels were also highest in PNZ (Figure 1H). Correlation analysis implied that M2 signature but not M1 signature were positive correlation with the hypoxic level in PNZ (Figure 1I). All these results indicated that M2 TAMs predicted poor prognosis for GBM patients and enriched in the PNZ of GBM.

### 3.2 Number of PGCCs associated with grade of glioma

**FIGURE 2:**
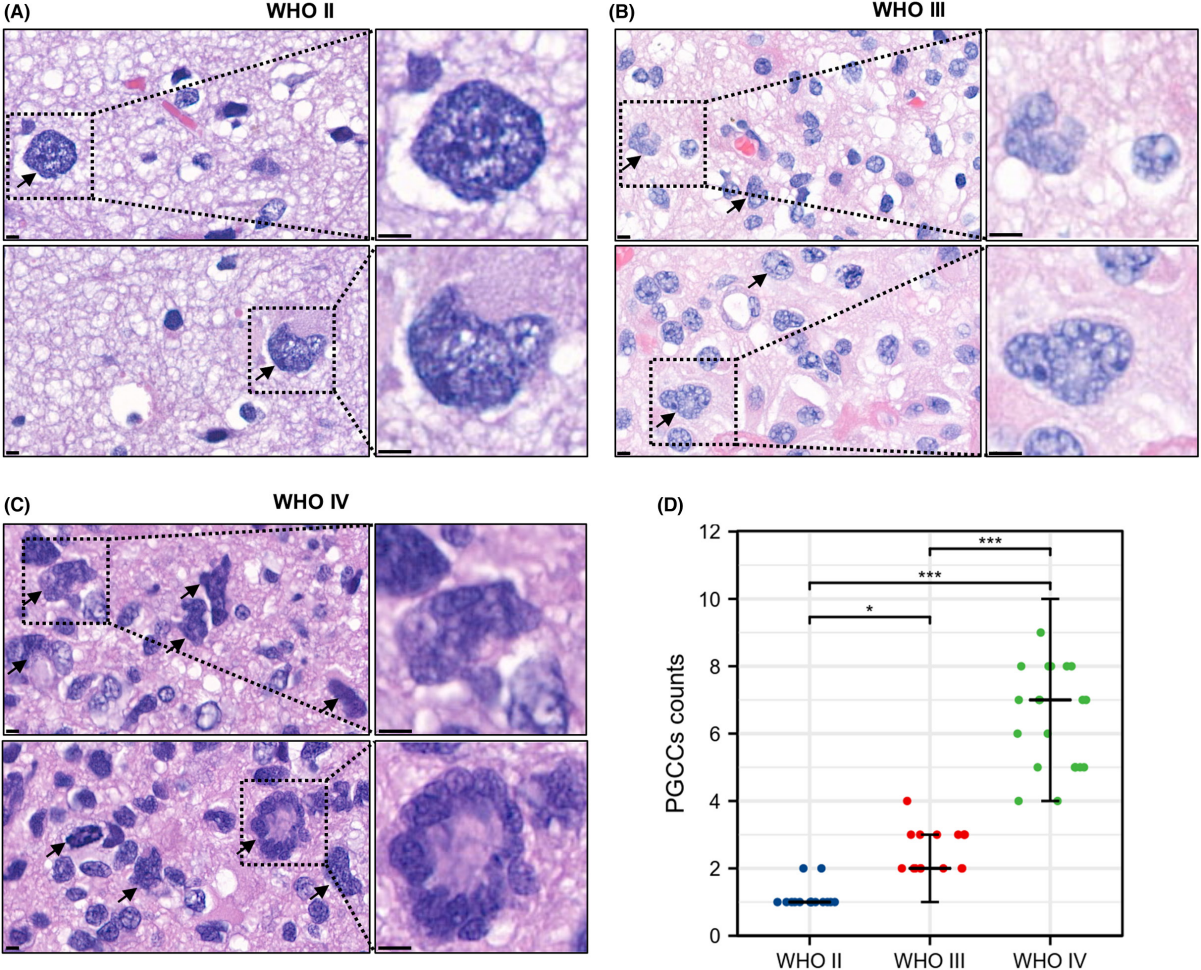
The numbers of PGCCs are associated with the grade of glioma. (A) Representative images of PGCCs in WHO grade II gliomas (the black arrow points a single PGCC, and the black frame shows a PGCC with single giant‐nucleus). (B) Representative images of PGCCs in WHO grade III gliomas (the black arrow points a single PGCC, and the black frame shows a PGCC with single giant‐nucleus). (C) Representative images of PGCCs in WHO grade IV gliomas (the black arrow points a single PGCC, and the lower black frame shows a PGCC with multi‐nucleus), Scale Bar: 5 μm. (D) Association between the number of PGCCs and the grade of gliomas. Data are shown as median ± interquartile range, *p < 0.05, *** *p* < 0.001, Kruskal–Wallis test and Dunn's multiple comparisons test

Using the characteristics of PGCCs set by Zhang et al.,^18^ results of maximal diameter measurements and morphologic observation indicated significant presence of PGCCs in all glioma tissues with different grades (Figure 2A–C). Additionally, PGCCs in high‐grade gliomas was more than those in low‐grade gliomas, and the differences were statistically significant (WHO II vs. WHO III, *p* = 0.023; WHO II vs. WHO IV, *p* < 0.001; WHO III vs. WHO IV, *p* = 0.001; Figure 2D). Morphologically, PGCCs exhibited either single giant‐nucleus (Figure 2A,B) or multi‐nucleus (Figure 2C). Furthermore, compared with diploid cells, the PGCCs nuclei displayed an irregular shape that suggested a high degree of atypia.

### 3.3 Generation of PGCCs under CoCl_2_ treatment

**FIGURE 3:**
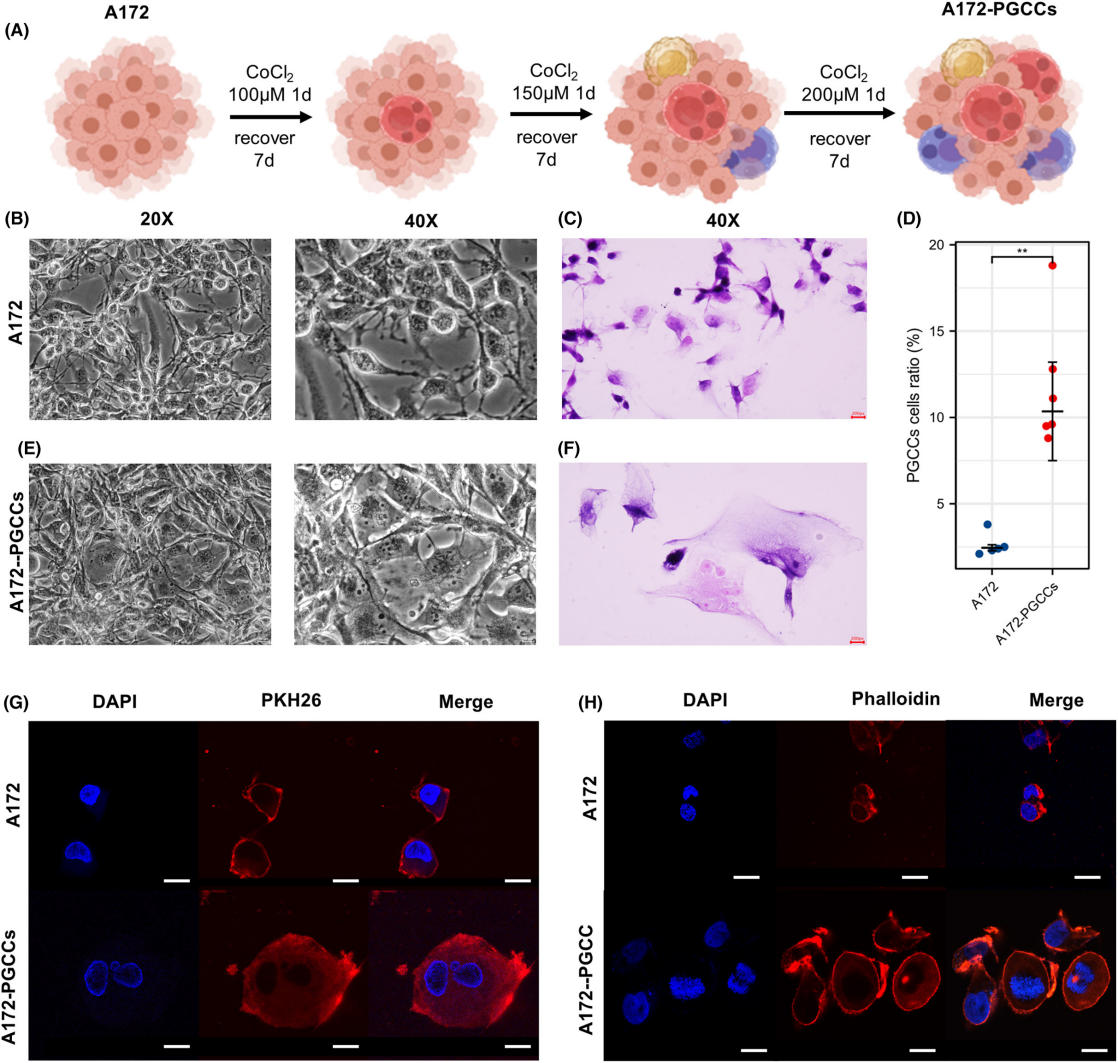
CoCl_2_ treatment induces PGCC formation in A172 cell cultures. (A) A schematic diagram shows the generation of PGCCs after consecutive treatment with CoCl_2_. (B, E) Morphological characterization of regular A172 cells and A172‐derived PGCCs in cultures before and after CoCl_2_ treatment (×20 and ×40). (C, F) H&E staining of regular A172 cells and A172‐derived PGCCs (×40). (D) Comparison of the proportions of PGCCs in A172 cell cultures without or with CoCl_2_ treatment. Data are shown as median ± interquartile range, **p < 0.01, Mann–Whitney U rank sum test. (G) Representative confocal microscopic images of double‐label immunofluorescent staining of PKH26 (red) and DAPI (blue) in A172 cell cultures without or with CoCl_2_ treatment. Scale Bar: 20 μm. (H) Representative confocal microscopic images of double‐label immunofluorescent staining of Phalloidin (red) and DAPI (blue) in A172 cell cultures without or with CoCl_2_ treatment. Scale Bar: 20 μm

To validate PGCCs' function in glioma, we used CoCl_2_, a hypoxia inducer, to treat the A172 cell line to generate the PGCCs (Figure 3A). Compared with original A172 cells, a giant skeleton presented in surviving cells, with acceleration in nucleus size or quantity in every cell. Following with accelerating concentration of CoCl_2_, those giant cells were more abundant in surviving cells. Under the continuous stimulation of high CoCl_2_ (200 μM), a domesticated phenomenon of giant‐nucleus or multi‐nucleus phenotype was stable in those cells, which could also be maintained for subsequent generations (Figure 3G and H). H&E staining of A172 and PGCCs slides confirmed that the proportion of PGCCs in the CoCl_2_‐treated group was significantly higher than that of the control group (*p* = 0.005, Figure 3D). Accompanied with characteristics of giant‐nucleus or multi‐nucleus, morphological observation detailed that the shape of PGCCs altered into round‐like from fusiform status, suggesting that PGCCs may undergo epithelial‐mesenchymal transition and obtain mesenchymal phenotype (Figure 3B, C, E, and F).

### 3.4 PGCCs produce daughter cells via the asymmetric division called neosis

**FIGURE 4:**
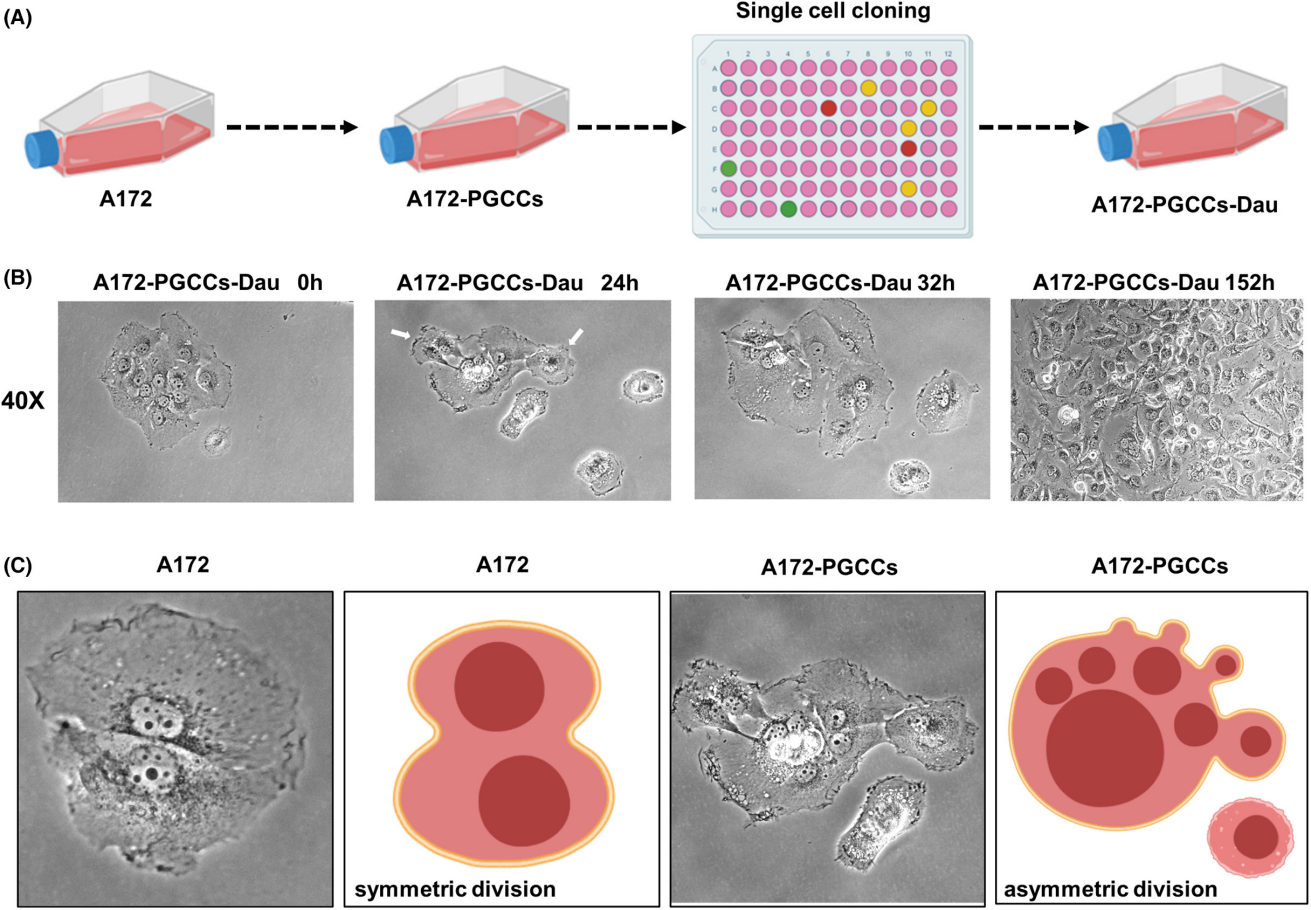
PGCCs produce daughter cells via the asymmetric division called neosis. (A) A schematic diagram shows the generation of A172‐PGCCs‐Dau via single‐cell cloning. (B) A single PGCC to form a colony at four consecutive time points. (C) Two different ways of cell division in cultures of A172 and A172‐derived PGCCs, respectively. As compared with A172 cells, PGCCs undergo asymmetric division

**FIGURE 5:**
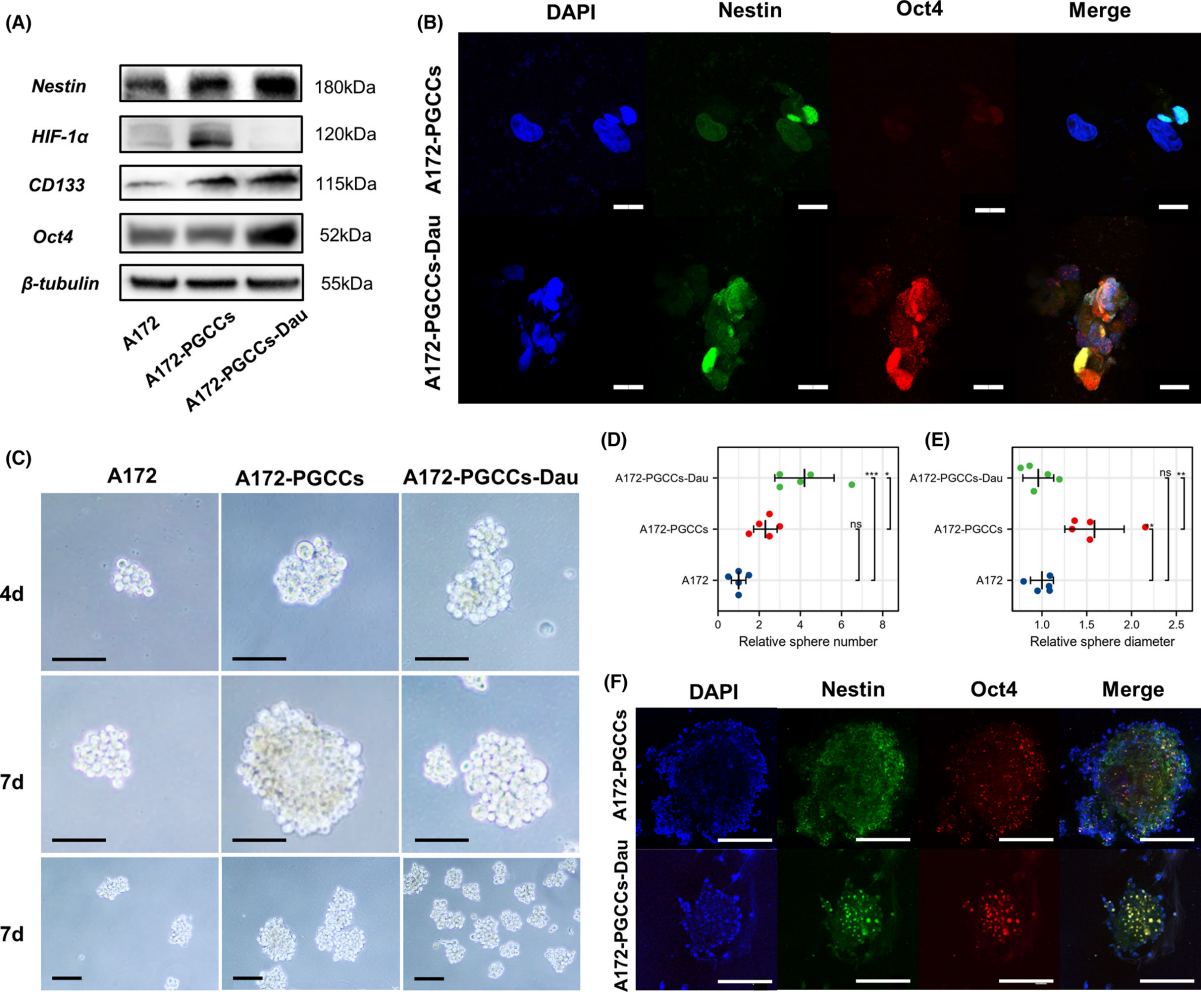
PGCCs and daughter cells acquire stemness. (A) Expression of stemness‐related proteins in A172, A172‐PGCCs, and A172‐PGCCs‐Dau. (B) Triple‐label immunofluorescence staining of Oct4, Nestin, and DAPI in cultures of A172, A172‐PGCCs, and A172‐PGCCs‐Dau. Scale Bar: 20 μm. (C) Representative light‐microscopic images of spheroids at 4 and 7 days in culture. Scale bars: 100 μm. (D) Relative sphere number. Data are shown as mean ± standard deviation, ns, *p* ≥ 0.05, *p < 0.05, *** *p* < 0.001, one‐way ANOVA and Tukey's multiple comparisons test. (E) Relative sphere diameter. Data are shown as mean ± standard deviation, ns, *p* ≥ 0.05, **p < 0.01, one‐way ANOVA and Tukey's multiple comparisons test. (F) Triple‐label immunofluorescence staining of Oct4, Nestin, and DAPI in cultures of spheroids derived from A172‐PGCCs and A172‐PGCCs‐Dau. Scale Bar: 100 μm

Based on the single‐cell cloning experiment, we obtained PGCCs‐Dau, the purified daughter cells of PGCCs (Figure 4A). A single multinuclear PGCCs cell was observed at four consecutive time points, which showed that PGCCs produced daughter cells through asymmetric division (Figure 4B). Compared with PGCCs, the daughter cells showed smaller sizes and fewer nuclei. These observations demonstrated that PGCCs might use a different manner to generate offspring than symmetrical division in A172 cells (Figure 4C). Because CoCl_2_ mimics hypoxia by stabilizing the hypoxia‐inducible transcription factor 1α (HIF‐1α), we examined the expression of HIF‐1α and found that HIF‐1α expression was markedly elevated in PGCCs relative to levels in A172 and PGCCs‐Dau (Figure 5A). The change of HIF‐1α expression level may suggest that A172 may adapt to the hypoxic microenvironment by producing PGCCs. When the hypoxic microenvironment eliminated, PGCCs began to produce progeny cells in order to reconstruct the cell population.

### 3.5 PGCCs and daughter cells acquire stemness

Previous studies have shown that PGCCs produced by other tumor cells have the characteristics of cancer stem cells.^17^, ^20^, ^30^ We speculate that PGCCs derived from GBM cell lines also acquired stemness. Western blotting results showed that compared with A172, the expression levels of stem cell markers (Nestin and CD133) increased in PGCCs and PGCCs‐Dau. Furthermore, the expression levels of Nestin, Oct4 and CD133 in PGCCs‐Dau were significantly higher than those in PGCCs, suggesting that PGCCs‐Dau may acquire stronger stemness than PGCCs (Figure 5A). Confocal analysis showed that PGCCs‐Dau exhibited higher expression levels of Oct4, consistent with the Western blotting results. Interestingly, the expression of Nestin was more robust in the small cells of PGCCs and PGCCs‐Dau, suggesting that these smaller‐sized cells may have stronger stemness (Figure 5B).

The spheroids formation assay was performed to further investigate the stemness of PGCCs derived from GBM (Figure 5C). The results showed that the relative sphere number was highest in PGCCs‐Dau in compared with A172 and PGCCs. (A172‐PGCCs‐Dau vs. A172‐PGCCs, *p* = 0.017; A172‐PGCCs‐Dau vs. A172, *p* < 0.001; A172‐PGCCs vs. A172, *p* = 0.104; Figure 5D). Moreover, the relative sphere diameter was highest in PGCCs in compared with A172 and PGCCs‐Dau. (A172‐PGCCs vs. A172‐PGCCs‐Dau, *p* = 0.003; A172‐PGCCs vs. A172, *p* = 0.004; A172‐PGCCs‐Dau vs. A172, *p* = 0.956; Figure 5E). The confocal microscopy analysis demonstrated that spheroid derived from PGCCs‐Dau were smaller but had higher expression levels of Oct4 and Nestin than that in PGCCs. All these results suggested that PGCCs‐Dau may acquire stronger stemness and different expression patterns of stemness in compared with PGCCs. (Figure 5F).

### 3.6 Daughter cells of PGCCs promotes the polarization of TAMs into M2 type

**FIGURE 6:**
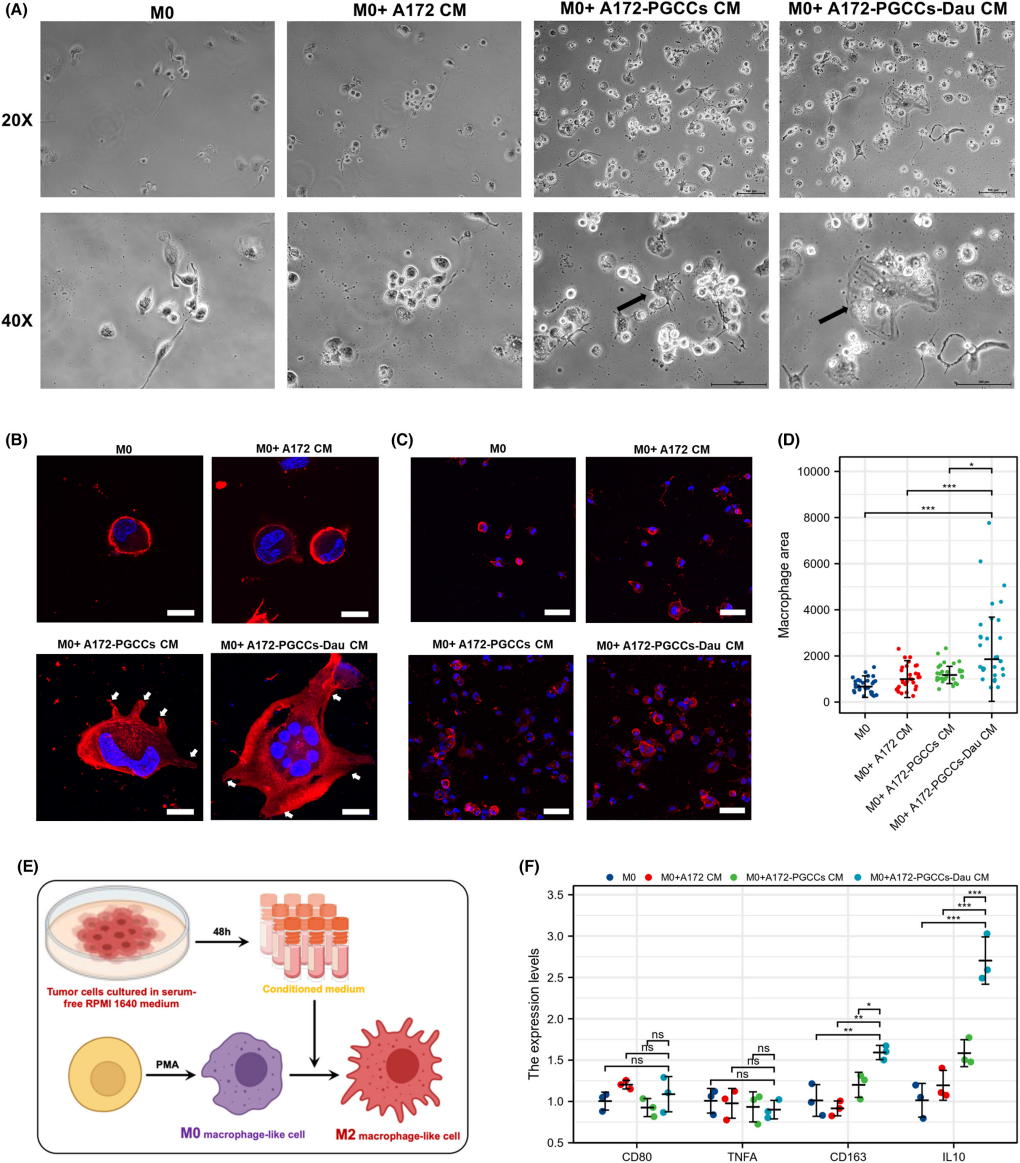
Daughter cells of PGCCs promote the polarization of TAMs to an M2 phenotype in vitro. (A) Representative light‐microscopic images of macrophages under different culture conditions (×20 and ×40). (B) Immunofluorescence staining of Phalloidin in macrophages under different culture conditions, the pseudopodia are arrowed (white arrowhead). Scale Bar: 20 μm. (C) Representative confocal microscope images of Phalloidin (red) and DAPI (blue) in cultures of M0 macrophages or M0 macrophages incubated with different conditioned medium. Scale Bar: 100 μm. (D) Comparison of cell area of macrophages in four groups. Data are median ± interquartile range, *p < 0.05, *** *p* < 0.001, Kruskal‐wallis test and Dunn's multiple comparisons test. (E) A schematic diagram shows the conditioned medium's preparation and the polarization of THP1 monocytes into M2 macrophage‐like cells. (F) qPCR analysis of expression of M2 markers (CD163 and IL10) and M1 markers (CD80 and TNFA). Data are mean ± standard deviation, ns, *p* ≥ 0.05, *p < 0.05, **p < 0.01, *** *p* < 0.001, one‐way ANOVA and Tukey's multiple comparison test

The above results demonstrated a spatial correlation between M2‐TAMs and PGCCs in PNZ of GBM, consistent with the literatures.^4^, ^10^, ^18^ For further exploration, we designed a co‐culture experiment to evaluate the effect of PGCCs on the macrophage. M0 macrophages generated from PMA‐stimulated THP1 cells were cultured in conditional culture media for 72 h. The structural changes were assessed using light microscopy. Compared to control groups, M0 macrophages treated with culture media from PGCCs or PGCCs‐Dau presented higher adherent ability, followed by larger sized and higher structural heterogeneity, indicating that culture media derived from PGCCs or PGCCs‐Dau promoted the survival of macrophages. Moreover, there were more obvious pseudopodia occurred, indicating that culture media derived from PGCCs or PGCCs‐Dau enabled more robust activation of M0 macrophages (Figure 6A). Besides, morphological detection by confocal fluorescent microscopy suggested that culture media from A172 promoted short pseudopodia. Comparatively, PGCCs or PGCCs‐Dau derived culture media lead to more abundant and outstretched pseudopodia, defined as filopodia, indicating more robust skeleton remodeling^31^ (Figure 6B,C). Moreover, quantification results of phalloidin staining revealed that the giant cells were generated under culture media from PGCCs‐Dau (M0 + A172‐PGCCs‐Dau vs. M0, *p* = 0.004; M0 + A172‐PGCCs‐Dau vs. M0 + A172 CM, *p* = 0.0001; M0 + A172‐PGCCs‐Dau vs. M0 + A172‐PGCCs, *p* = 0.0252; Figure 6D). Further qRT‐PCR results suggested that the expression levels of M2‐type macrophage markers (CD163 and IL‐10) were increased in macrophages treated with PGCCs‐Dau conditioned medium (CD163: M0 + A172‐PGCCs‐Dau CM vs. M0, *p* = 0.004; M0 + A172‐PGCCs‐Dau CM vs. M0 + A172 CM, *p* = 0.001; M0 + A172‐PGCCs‐Dau CM vs. M0 + A172‐PGCCs CM, *p* = 0.034; IL10: M0 + A172‐PGCCs‐Dau CM vs. M0, *p* < 0.001; M0 + A172‐PGCCs‐Dau CM vs. M0 + A172 CM, *p* < 0.001; M0 + A172‐PGCCs‐Dau CM vs. M0 + A172‐PGCCs CM, *p* = 0.001). However, there was no significant difference in markers of CD80 and TNF‐α in M1‐type macrophages (Figure 6F). These results suggest that PGCCs‐Dau can induce M2 polarization of macrophages. A schematic diagram of the experiment can be seen in Figure 6E.

## 4 DISCUSSION

Previous studies have unearthed the role of GSCs in the poor outcome of GBM patients.^15^ GSCs may promote malignant proliferation, angiogenesis, and therapeutic resistance in various ways.^4^, ^23^ Another significant characteristic of GSC is forming a unique cell niche to reform the intratumor microenvironment, which conversely results in a more robust evolution of GSCs.^11^ Therefore, it is essential to screen or define the GSCs. Furthermore, the crosstalk between GSCs and the microenvironment is also under the control of multiple types of cells, high heterogeneity of GSCs origin and phenotype making it more complicated.^9^ Currently, there is a lack of clear stem evaluation indicators and corresponding evaluation criteria.^32^

In recent years, PGCCs, a unique subtype of GSCs, have demonstrated high stemness and plasticity and extensively participated in the malignant behaviors of many solid tumors. The first evidence is that specific marker genes of stemness are enriched in PGCCs. Zhang et al. reported the elevated CD133 and CD44, the classical stem cell markers, expressed on the daughter cells of PGCCs.^20^ PGCCs derived from Mullerian epithelial or ovarian cancer cells are also marked with increased stemness markers such as Oct4, Nanog, and Sox‐2.^30^, ^33^ The stem cell characteristics endow PGCCs with multidirectional differentiation potentials. PGCCs induced by paclitaxel from ovarian cancer cells can produce various offsprings, including epithelial cells, fibroblasts, neuronal cells, and yeast‐like cells.^30^ In addition, PGCCs derived from ovarian cancer cells can also differentiate into fat, cartilage, and bone tissue.^20^ In breast and rectal cancer studies, PGCCs have been proved to produce red blood cells. The above results suggest that PGCCs have a remarkable ability of multidirectional differentiation.^33^ In addition, Zhang et al. showed that a single PGCCs cell derived from tumor cells can spheroidize in vitro and tumorigenesis in vivo in immunodeficient mice.^20^

Various stresses, such as chemotherapy, radiotherapy, and hypoxic microenvironment, can induce PGCCs from diploid tumor cells.^34^ Hypoxia is a leading feature of the GBM tumor microenvironment and plays an essential role in stemness induction and maintenance. Moreover, it can also promote GSCs to mediate tumor recurrence and drug resistance.^11^, ^35^ Our current study confirms that PGCCs could be directly induced from a GBM cell line with CoCl_2_, a chemical hypoxic simulator. PGCCs exhibit two different types of nuclei compared with parental cells: single macronucleus and multinucleus, which is in line with the high heterogeneity of GBM‐PGCCs. Shu et al. proposed that during the cell division process of PGCCs, multi‐nuclear PGCCs are more likely to be produced when nuclear replication and separation are completed, and cytoplasmic separation fails. On the contrary, if nuclear and cytoplasmic separation fails at the same time, single large nuclear PGCCs would appear.^36^

PGCCs employ asymmetric division to produce daughter cells (PGCCs‐Dau); almost all cells are small and mononuclear.^20^ Rajaraman et al. described the way of PGCCs to produce progeny cells as “neosis,” which is different from mitosis and meiosis, and the progeny cells are named with “Raju cells” or “escape cells.”^37^ Meanwhile, the asymmetric division causes mononuclear progeny cells to lose some genetic material, leading to higher genetic instability and heterogeneity.^34^ Compared with their parental PGCCs, the progeny cells of PGCCs acquire distinct characteristics. Through spectral karyotype analysis, Niu et al. found that the progeny cells derived from PGCCs obtained a new genome. Compared with parental HEY and SKOV3 cells, chromosome recombination and genomic changes appeared in progeny cells.^19^ Additionally, progeny cells of PGCCs derived from ovarian cancer are more resistant to apoptosis and chemotherapy than diploid cells.^30^ In the study of colorectal cancer PGCCs, it was found that the progeny cells showed more robust migration and invasion ability than diploid cells, which could promote lymph node metastasis by expressing EMT‐related proteins.^38^ Zhang et al. revealed that PGCCs play a central role in tumor development and progression under hypoxic microenvironment conditions. PGCCs with their daughter tumor cells, and erythroid cells could form vasculogenic mimicry to promote tumor penetration and metastasis.^34^, ^38^, ^39^ Hence, it is reasonable to consider that PGCCs may have intimate relationships with neovascularization and oxygen metabolism in GBM. These “giant monsters” and their daughter cells might acquire aggressive phenotype and unique energy metabolism to facilitate invasion and recurrence of GBM. Our study showed that GBM derived PGCCs expressed stemness markers. Moreover, the stemness can also be delivered and even augmented in progeny cells. The above results suggest that PGCCs enable GBM cells to tolerate hypoxia and may further mediate GBM recurrence and therapeutic resistance.

Besides directly affecting GBM recurrence or treatment resistance, PGCCs may promote malignancy by altering the inhibitory immune tumor microenvironment. M2 TAMs are a specific type of activated macrophage, playing an important role in supporting GBM malignant proliferation and progression.^40^, ^41^, ^42^ As we performed in this study, high infiltration of M2 TAMs is closely linked with poor outcomes in GBM patients. GSCs can regulate the recruitment, polarization, and survival of TAMs in different ways. For example, GSCs promote TAMs recruitment in GBM by secreting OLFML3, POSTN, CXCL12B, CCL5, and CXCL1. GSCs can also promote M2 polarization in GBM through a STAT3‐dependent way.^9^ Tao et al. found that GSCs secrete the WISP1 protein regulation integrin α6β1‐Akt pathway, which promotes the survival of M2 TAMs.^4^ Combined with bioinformatics analysis and literature research, PGCCs and TAMs are co‐located and enriched in the hypoxic region (PNZ) around the necrotic focus in GBM tumor.^10^, ^18^ The results of our co‐culture assay revealed that PGCCs‐Dau exsert the most potent ability to activate macrophages and promote the polarization of macrophages to M2 type. Bharadwaj et al. demonstrated that PGCCs have a unique senescence‐associated secretory phenotype (SASP), including various chemokines and growth factors that can act on macrophages.^43^ These scientific inventions laid the foundation of the immune‐modulatory of PGCCs on activation of macrophages in GBM; further studies are needed to unearth the mechanism.

There are several limitations to the current study. First, we carried out the majority of assays using the A172 cell line. To further clarify the biological role of PGCCs in glioma, we should perform the same analyses using other cell lines in future studies. Second, although we used the hypoxic region of GBM as the link for TAMs and PGCCs, there is a lack of investigation for co‐localization analysis based on glioma specimens. Third, this study mainly focused on the influence of PGCCs on TAMs, whereas the effect of TAMs on PGCCs also warrants in‐depth investigation to illustrate the crosstalk between them.

## 5 CONCLUSION

In conclusion, our study revealed that hypoxia‐induced PGCCs have stemness properties, which are even more profound in their daughter cells. PGCCs daughter cells can promote inhibitory immune microenvironment by inducing M2 polarization of macrophages. This study provided a new perspective that PGCCs and their offsprings might be a promising therapeutic target for treating GBM patients.

## AUTHOR CONTRIBUTIONS

Yuyang Liu, Chuan Xu and Ling Chen contributed to planning the study. Mengwan Wu and Jialin Liu performed data collection and analysis. Ying Shi and Hong Wu were responsible for the data interpretation. Yuyang Liu and Ying Shi drafted and revised the manuscript. Chuan Xu and Ling Chen revised. All authors read and approved the final version of the manuscript.

## CONFLICT OF INTEREST

The authors declare no conflict of interest.

## Supporting information


**Table S1** Antibodies used in this study

**Table S2** Primers used for qPCR analyses in this study

## Data Availability

All data associated with this study are present in the paper or the Supplementary Materials.
