# Supplementary material for: Hypoxia‐induced polypoid giant cancer cells in glioma promote the transformation of tumor‐associated macrophages to a tumor‐supportive phenotype
Source: CNS Neurosci Ther. 2022 Jun 28;28(9):1326–38. doi: 10.1111/cns.13892 (PMC9344088; doi:10.1111/cns.13892)
Supplement: Supplementary file 1 — Table S1 Antibodies used in this study [file CNS-28-1326-s002.docx]

**Supplementary Table 1. Antibodies used in this study**

| Antibody | Source | Supplier | Dilution |
| --- | --- | --- | --- |
| Anti-Oct4 | Mouse | PROTEINTECH (60242-1-Ig) | 1:2000 (WB)  1:500 (IF) |
| Anti-Nestin | Rabbit | ABCAM (ab105389) | 1:2000 (WB)  1:500 (IF) |
| Anti-CD133 | Mouse | PROTEINTECH (66666-1-Ig) | 1:2000 (WB) |
| Anti-HIF1α | Rabbit | PROTEINTECH (20960-1-AP) | 1:2000 (WB) |
| Anti-Beta Tubulin | Rabbit | PROTEINTECH (10094-1-AP) | 1:3000 (WB) |

IF, immunofluorescence; WB, Western blotting
