# Supplementary material for: Hypoxia‐induced polypoid giant cancer cells in glioma promote the transformation of tumor‐associated macrophages to a tumor‐supportive phenotype
Source: CNS Neurosci Ther. 2022 Jun 28;28(9):1326–38. doi: 10.1111/cns.13892 (PMC9344088; doi:10.1111/cns.13892)
Supplement: Supplementary file 2 — Table S2 Primers used for qPCR analyses in this study [file CNS-28-1326-s001.docx]

**Supplementary Table 2. Primers used for qPCR analyses in this study**

| Gene | Forward primer (5’ to 3’) | Reverse primer (5’ to 3’) |
| --- | --- | --- |
| CD163 | GTCGCTCATCCCGTCAGTCATC | GCCGCTGTCTCTGTCTTCGC |
| IL-10 | GGCACCCAGTCTGAGAACAG | ACTCTGCTGAAGGCATCTCG |
| CD80 | AAACTCGCATCTACTGGCAAA | GGTTCTTGTACTCGGGCCATA |
| TNFA | ATCTTCTCGAACCCCGAGTGA | CGGTTCAGCCACTGGAGCT |
| GAPDH | GAAGGTGAAGGTCGGAGTC | GAAGATGGTGATGGGATTTC |
